# Mouse Models for Blistering Skin Disorders

**DOI:** 10.1155/2010/584353

**Published:** 2010-05-10

**Authors:** Radhika Ganeshan, Jiangli Chen, Peter J. Koch

**Affiliations:** ^1^Department of Dermatology, University of Colorado Medical School, 12800 East 19th Avenue, Aurora, CO 80045, USA; ^2^Department of Cell and Developmental Biology, University of Colorado Medical School, Aurora, CO 80045, USA

## Abstract

Genetically engineered mice have been essential tools for elucidating the pathological mechanisms underlying human diseases. In the case of diseases caused by impaired desmosome function, mouse models have helped to establish causal links between mutations and disease phenotypes. This review focuses on mice that lack the desmosomal cadherins desmoglein 3 or desmocollin 3 in stratified epithelia. A comparison of the phenotypes observed in these mouse lines is provided and the relationship between the mutant mouse phenotypes and human diseases, in particular pemphigus vulgaris, is discussed. Furthermore, we will discuss the advantages and potential limitations of genetically engineered mouse lines in our ongoing quest to understand blistering skin diseases.

## 1. Introduction

In recent years, mouse models have become an essential tool for studying genetic diseases, especially in cases where the disease is caused by mutations in a single gene (monogenic disorders). It has been a challenge, however, to faithfully reproduce the pathophysiology of autoimmune disorders in mice, in part because the human disease is influenced by complex factors such as the genetic background, the nature of the autoantibodies (epitopes recognized and antibody subclass) and titers of the circulating autoantibodies. 

Pemphigus is a class of autoimmune blistering skin diseases which manifest itself in the form of painful lesions in the skin and oral mucosa and which, in the case of pemphigus vulgaris, can be lethal if left untreated. The sera of patients with pemphigus contain autoantibodies directed against desmosomal cadherins (e.g., desmogleins and most likely desmocollins; see below) [1, 2], a group of transmembrane glycoproteins that are required to establish and maintain cell-cell adhesion between epidermal keratinocytes (reviewed in [3]). Desmogleins and desmocollins are transmembrane glycoproteins that are thought to establish cell coupling via binding of their extracellular domains. On the cytoplasmic surface of the plasma membrane, these cadherins are connected to the intermediate filament cytoskeleton via a network of desmosomal plaque proteins (desmoplakin, plakoglobin, plakophilins).

How does the binding of pathogenic pemphigus autoantibodies induce intraepithelial blistering, the characteristic histopathological feature of the disease? Do these antibodies inhibit the function of these proteins? A simple approach to test this hypothesis is to eliminate the target protein in genetically engineered mice and to determine whether the resulting loss-of-function phenotype replicates the disease. 

Pemphigus vulgaris (PV) patients develop autoantibodies that target desmoglein (DSG) 3 and under certain conditions desmoglein (DSG) 1 [1]. Patients with DSG3 antibodies alone develop mucous membrane lesions whereas, in the presence of both DSG3 and DSG1 antibodies, skin lesions are observed as well [1]. Furthermore, it has been shown that injecting DSG3-specific PV antibodies into newborn mice can replicate the histopathology of the disease, demonstrating the pathogenicity of these antibodies (see [1]).

Based on the assumption that autoantibodies neutralize adhesive functions, one would predict that loss of *Dsg3* function in mice would mimic the phenotype of PV restricted to mucous membranes. To test this hypothesis, we generated *Dsg3* null mice.

## 2. Null Mutation in *Dsg3* Provided First Functional Link to PV

Mice with a targeted disruption of *Dsg3* exhibited phenotypes very similar to those seen in PV patients and provided direct evidence for a role of DSG3 in maintaining cell-cell adhesion between keratinocytes. A hallmark feature of PV in humans is acantholysis (loss of cell-cell adhesion) just above the basal layer in stratified epithelia. Furthermore, basal keratinocytes often lose lateral cell contact, leading to a histological finding that has been called a “row of tombstones”. The *Dsg3*-null mice developed severe erosions of the oral mucosa (similar to that seen in PV patients) that prevented them from feeding thus causing runting. Suprabasal blistering was also evident in other stratified epithelia such as the vaginal epithelium (see below). Overt skin lesions were not noted in these mice, except for areas exposed to significant mechanical stress such as the skin around the snout, the nipples of nursing females, and the muco-cutaneous junctions in the eyes [4, 5]. A histological examination of other mouse tissues that express DSG3, such as the esophagus, the forestomach (which is lined by a stratified epithelium structurally similar to the epidermis), and the thymus, did not reveal abnormalities. Note that the forestomach is a characteristic feature of the mouse which is not present in humans.

**Figure 1:**
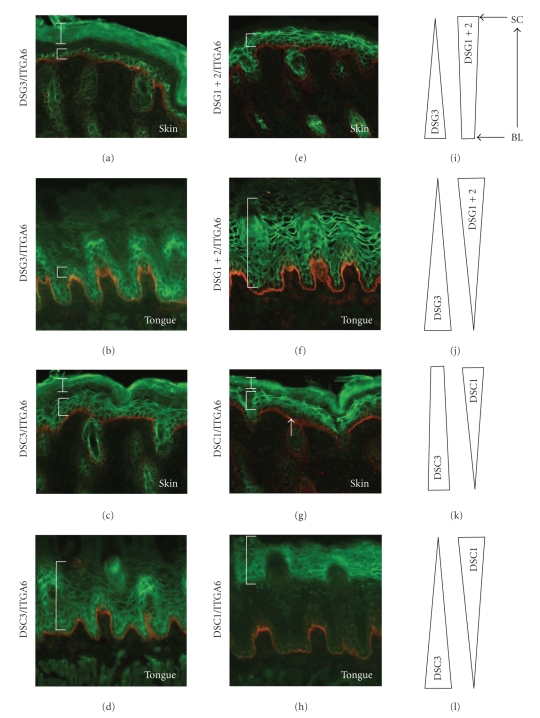
Expression of the desmosomal cadherins desmoglein 1, 2, and 3 (DSG1–3) and desmocollin 1 and 3 (DSC1, 3) as well as *α*6-integrin (ITGA6) in the interfollicular epidermis and the tongue epithelium of mice: Immunofluorescence staining of newborn epidermis ((a), (e), (c), (g)) and adult tongue ((b), (d), (f), (h)). The cadherins are shown in green while the integrin is shown in red. ((i), (j), (k), (l)) Schematic representation of the distribution of each desmosomal cadherin in the epidermis ((i), (k)) and tongue epithelium ((j and l)). Note that DSC2 antibodies which recognize the mouse isoform are currently not available (see text for details). Immunofluorescence signals from the stratum corneum are due to nonspecific binding of secondary antibodies (*white bar*). Cell layers expressing the relevant proteins are marked with brackets. The white arrow in (g) points towards the basal layer, which does not synthesize DSC1. Note that the expression of DSG1 + 2 in the tongue is low in the basal layers. (i, j, k, l) Distribution of the desmosomal cadherins in stratified epithelia (BL, basal layer; SC, stratum corneum). High expression levels are symbolized by a broad base and low expression levels are symbolized by narrow tips.

A likely explanation for the absence of lesions in these tissues lies in the expression of functionally redundant desmoglein isoforms which compensate for the loss of DSG3. This idea is consistent with the compensation hypothesis, put forward by John Stanley and colleagues (see [2]), to explain the tissue-specificity of pemphigus autoantibodies. Based on this idea, one would predict that DSG3 is the predominant or the only DSG isoform expressed in the area where acantholysis occurs (e.g., in the deep layers of the oral mucosa). Further, extensive overlap between DSG3 and other DSG isoforms should exist in tissues and cell layers where no spontaneous lesions were detected. The distribution of the three major DSG isoforms (DSG1–3) in affected (mucous membranes) and unaffected tissues (e.g., skin) appears to be largely consistent with this idea as illustrated in Figure 1. In the skin of mice, DSG3 is restricted to the basal and immediate suprabasal cell layers, whereas DSG1 and 2 are present throughout the epithelium. Thus, DSG1 and 2 ensure that cell-cell adhesion is maintained in *Dsg3* null skin (Figure 1). Nevertheless, traumatizing the skin by rubbing or scratching resulted in lesions, indicating that in the absence of DSG3, the mechanical strength of the epithelial tissue was compromised. In mucous membranes, DSG1 and 2 are present throughout the epithelium but are weaker in the deep epidermis where DSG3 dominates as judged by immunohistochemical staining (Figure 1(b)), thus explaining the PV-like acantholysis observed in *Dsg3* null oral mucosa.

## 3. Loss of *Dsc3* and *Dsg3* Lead to Similar Histopathology but Target Different Tissues

Desmosomal adhesion is thought to be mediated both by homophilic as well as by heterophilic interactions between desmogleins and desmocollins, which form the adhesive core of desmosomes (e.g., [6, 7]). The relative contributions of homophilic (DSG-DSG; DSC-DSC) and heterophilic (DSG-DSC) interactions to establish and maintain cell adhesion are currently not known. In the case of DSG3, it had originally been speculated that a DSG3-DSC3 complex might be essential to maintain keratinocyte adhesion in the deep layers of stratified epithelia (for an alternative view see [6]). We thus hypothesized that ablating *Dsc3* in mice might mimic the effects of loss of DSG3, leading to PV-like histopathology. 

**Figure 2:**
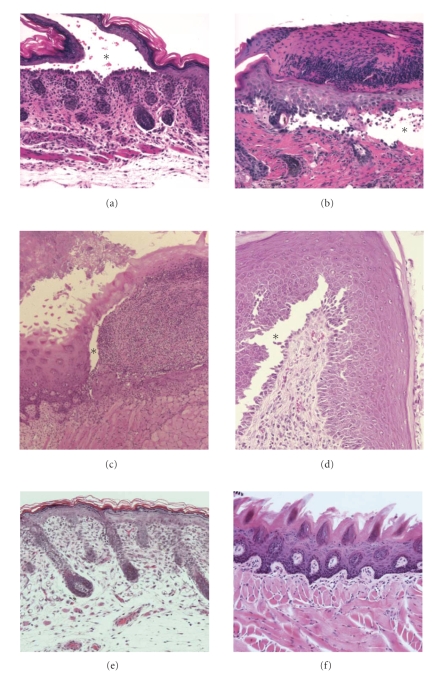
Acantholysis in the interfollicular epidermis and mucous membranes of *Dsc3* and *Dsg3* null epithelia. (a) Acantholysis between the basal and first suprabasal layer in the back skin epidermis of newborn conditional *Dsc3* null mice (*Dsc3^fl/fl^*/K14-Cre). (b) Severe skin lesions of a 140 day-old *Dsc3^fl/fl^*/K14-Cre mouse showing blistering in a healing wound. Note that these mice enter a cycle in which acantholysis triggers epithelial tongue formation and secondary blistering in the epithelium that covers the original wound. (c) Tongue section from a 25-day-old *Dsg3* null mouse showing acantholysis and massive inflammation in the epithelium. (d) Vagina of a 6-month-old *Dsg3* null mouse showing acantholysis in the deep epithelium (between basal and suprabasal layer). (e) Back skin of an adult wild type mouse. (f) Tongue histology of an adult wild type mouse. Stars indicate blister cavities.

Since germline deletion of the *Dsc3* gene was embryonic lethal [8], conditional *Dsc3* null mutant mouse lines were generated that lacked DSC3 expression in basal keratinocytes of stratified epithelia, including skin and mucous membranes [9]. These tissue-specific *Dsc3* null mice developed skin blisters (Figures 2(a) and 2(b)) that were histologically similar to those found in humans with muco-cutaneous PV. In contrast to skin lesions in *Dsg3*-null mice (which were restricted to areas exposed to significant mechanical trauma), *Dsc3* null skin blisters developed spontaneously and were present in all skin samples of newborn mice that were analyzed. Nevertheless, the extent of blister formation varied between individual mice, most likely due to different degrees of mechanical stress to which the animals were exposed prior to tissue harvesting. Even small trauma resulted in extensive skin blistering in *Dsc3* null skin, a typical characteristic of PV in humans (Nikolsky's sign). 

Unlike the lesions observed in *Dsg3*-null mutants (Figures 2(c) and 2(d)), lesions in the *Dsc3* null mice were restricted to the skin and were not present in internal stratified epithelia, such as those of the oral cavity. As in the case of the *Dsg3*-null mice, we believe that the restriction of the blistering phenotype to the skin can be explained by the expression of compensatory proteins, in this case DSC isoforms. In the skin, DSC3 is present throughout the epithelium with weaker expression levels in the granular layers (Figure 1). DSC1 is mainly restricted to the granular cell layer of the interfollicular epidermis. The distribution of DSC2 in the mouse epidermis is currently not known, due to the lack of antibodies that recognize the mouse isoform. Nevertheless, in humans and cows, it is known that *Dsc2* is only weakly expressed or even absent in most of the interfollicular epidermis, with the notable exception of the palms and soles (see [9]). Given that the DSC1 and DSC3 expression patterns are very similar in mice and humans, it is reasonable to speculate that the distribution of DSC2 is also very similar in both species. Consequently, DSC3 would be the major DSC isoform expressed in the deep layers of the epidermis, thus explaining why loss of *Dsc3* causes acantholysis in these cell layers. How to explain the absence of lesions in the oral cavity of *Dsc3* mutants? We believe that the key to understanding the tissue specificity of these lesions is the distribution of DSC2. Data from bovine samples indicated that *Dsc2* is strongly expressed throughout all layers of internal stratified epithelia, such as tongue [10–13]. The combination of DSC1 and DSC2 is thus likely to maintain cell-cell adhesion in oral mucosa in the absence of DSC3 (Figure 1, and data not shown). Nevertheless, little is known regarding the molecular (homophilic and heterophilic) interactions between desmosomal cadherins in vivo; that is, other models of compensation are possible. Given that homophilic interactions between desmosomal cadherins might occur in vivo, it is also possible that DSG3-DSG3 interactions alone might be sufficient to maintain cell adhesion in the *Dsc3* null mucosa.

The data summarized above suggest that DSG3 and DSC3 have comparable functions in desmosomes. However, they have different roles in cell adhesion of specific tissues, due to the presence of different compensatory proteins in different tissues. Thus, whereas DSG3 appears to be essential for maintaining cell-cell adhesion of internal stratified epithelia, DSC3 plays a similar role in the epidermis of the skin.

## 4. Null Mutations in Desmosomal Cadherins Cause Hair Loss

**Figure 3:**
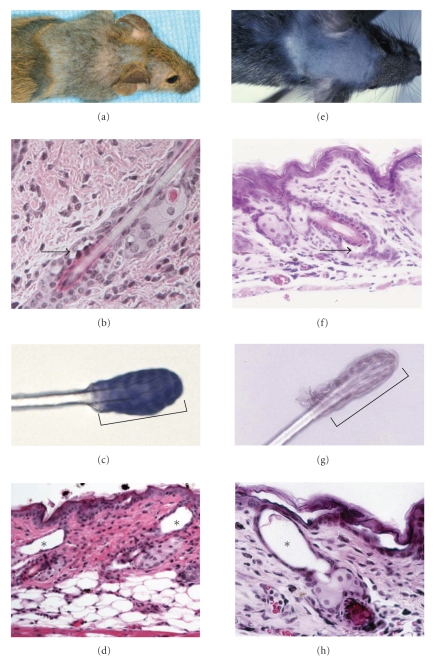
Hair loss phenotype of conditional *Dsc3* null ((a), (b), (c), (d)) and conventional *Dsg3* null mice ((e), (f), (g), (h)). Both mouse lines show cyclic hair loss beginning around the time of weaning when the hair follicles on the back of the head enter telogen, the resting phase of the hair growth cycle. (a) Hair loss of a 56-day-old conditional *Dsc3* null mouse and (b) a 53-day-old *Dsg3 *null mouse. (b and f) Acantholysis between the two cell layers surrounding the telogen hair club leads to loss of the hair shaft. Arrows indicate separation of the two cell layers. *Note that early lesions are shown, that is, before the hair shaft is actually lost*. (c and g) Telogen hairs with a single epithelial cell layer surrounding the club hair (brackets, nuclei of the epithelial sheet surrounding the clubs are stained in dark (c) and light (g) blue, resp.). In both cases, hair loss leads to the development of dermal cysts ((d and h); stars). Note that the hair loss in both mouse lines appears to follow the same mechanism, that is, loss of cell-cell adhesion between the two epithelial cell layers which anchor the telogen hair in the skin.

Interestingly, both desmosomal cadherins are required to anchor hair follicles in the skin, as demonstrated by the cyclic hair loss observed both in *Dsg3* null and *Dsc3* null mice (Figure 3). The hair loss in both mutants was initiated around the time of weaning and progressed from the top of the head to the tail. This hair loss was never observed in wild type litter mates, that is, this phenotype was strictly linked to the null mutations in the two desmosomal cadherin genes. Further, loss of telogen hair, as demonstrated by tape hair-stripping experiment (Figures 5(c) and 5(g)), was observed only in the mutant animals and not in wild type littermates. Histology of the bald areas of the skin revealed intraepithelial blistering affecting the two keratinocyte cell layers surrounding the telogen club hair (Figures 3(b) and 3(f)). Acantholysis led to a loss of the hair shaft followed by formation of empty dermal cysts (Figures 3(d) and 3(h)). Nevertheless, the hair in these animals grew back and was lost again in the next telogen phase. This cycle repeated itself several times. Eventually, some of the older mice remained bald, possibly due to hair follicle stem cell depletion.

The patterned loss of hair is due to synchronization of the hair cycle in mice; around the time of weaning hair follicles progress from the first postnatal anagen (the active phase of hair growth cycle) to telogen (resting phase of the hair growth cycle). Further, the wave of hair follicle cycling proceeds from head to tail of the mouse. In rare instances, hair loss has been reported in PF and PV patients [5, 14], suggesting that the mouse models discussed here mimic a human disease phenotype. However, due to the synchronization of the mouse hair cycle (which does not occur in humans), this effect is much more severe in desmoglein and desmocollin null mice than in humans with the autoimmune disease.

## 5. Dissecting the Roles of Desmosomal Cadherins in Cell Adhesion, Signaling, and Skin Disorders

The *Dsg3* null mice do not develop an autoimmune disease; that is, they do not provide us with a tool to dissect autoantibody-mediated pathology. Nevertheless, these mice enabled us for the first time to link the loss of DSG3 function to PV histopathology. In other words, we were able to functionally identify DSG3 as a likely key target in a subset of pemphigus patients. Subsequent to the development of the *Dsg3*-null mouse model, Amagai and colleagues utilized this mouse line to develop an autoimmune model for PV in mice [15], demonstrating the usefulness of genetically engineered mouse models for understanding the molecular pathology of human diseases. Nevertheless, the mechanism by which PV autoantibodies induce loss of DSG3 function and cause PV is still a controversial issue. The hypotheses range from direct inhibition of desmoglein function to indirect loss of cell-cell adhesion. For instance, PV antibodies appear to trigger aberrant intracellular signaling in several pathways. Several reports have provided a link between pemphigus and abnormal signaling mediated by p38 MAPK pathway and by plakoglobin ([16, 17], see [3]). Based on studies that used cultured keratinocytes, it appears that pemphigus autoantibodies trigger phosphorylation of p38 MAPK which in turn induces acantholysis by affecting downstream effectors such as RhoA or HSP27 [16, 18]. Interestingly, inhibitors of p38 MAPK activation could prevent skin blister formation in newborn mice injected with PV autoantibodies (passive antibody transfer model for PV), which suggests that signaling mechanisms involving p38 MAPK are central to acantholysis in pemphigus. Results from another group implicated depletion of the cellular plakoglobin pool leading to increased expression of c-Myc to be critical for PV pathogenesis [17, 19]. Although the role of c-Myc signaling in acantholysis is not clearly understood, the fact that c-Myc inhibitors abrogated skin blistering induced by PV autoantibodies illustrates that this mechanism could be important in PV pathogenesis. It, therefore, appears that the mechanisms driving the loss of cell adhesion by impaired desmosomal function are more complex than previously believed. 

Although it has been well established that DSG3 plays a key role in the development of PV, less is known about a potential role of desmocollins in autoimmune diseases. DSC1 has been linked to IgA pemphigus (see [3]), whereas a role for DSC3 in autoimmune diseases has not yet been established. The phenotypic consequences of a *Dsc3* null mutation in stratified epithelia of mice suggest that loss of function of this protein, either due to mutations or due to autoantibodies, would result in PV-like lesions. Recent reports suggest that this might indeed be the case. Bolling and colleagues reported the case of a patient with PV-like symptoms who had developed DSC3- but not DSG3-autoantibodies [20]. This report did not establish, however, whether the DSC3-specific antibodies from this patient were pathogenic. Further, Ayub and colleagues recently reported a *DSC3* mutation in a family in Afghanistan that was associated with hair loss and recurrent skin blistering [21], that is, a phenotype strikingly similar to the phenotype of our *Dsc3* mutant mice. Nevertheless, this report did not provide histological data demonstrating intraepidermal blistering; that is, further evidence is required to convincingly link impaired DSC3 function to intraepidermal blistering in humans.

Taken together, our mouse study demonstrated that loss of *Dsc3* function can lead to PV-like lesions while the human studies cited above suggest that this finding is relevant for at least a subgroup of patients with PV-like disease. Further studies will be necessary to determine the extent to which DSC3 plays a role in inherited and acquired desmosomal diseases.

## 6. Conclusions

The last decade has seen a surge of information regarding the roles of individual desmosomal genes in normal development and diseases based on the analysis of transgenic and knockout mice. The ability to switch genes on or off in specific cell types and tissues at predetermined time points has made the mouse model a premier tool for discovering gene functions and for the elucidation of disease mechanisms. Moreover, the core genetic and physiologic pathways that control epithelial cell differentiation (including appendage development) are highly conserved between humans and mice. Although the ease of genetic manipulations and the resemblance to human physiology make mice an attractive choice to study skin disorders, there are potential pitfalls, such as the possibility of species-specific differences in the histology of the skin. Further, it is also not possible to exclude the existence of species-specific gene functions, requiring confirmation of experimental results in a human test system. Nevertheless, the mouse will remain a valuable tool for the analysis of human diseases and for the advancement in our understanding of basic epithelial cell biology.
